# Screening of Drug Repositioning Candidates for Castration Resistant Prostate Cancer

**DOI:** 10.3389/fonc.2019.00661

**Published:** 2019-07-23

**Authors:** In-Wha Kim, Jae Hyun Kim, Jung Mi Oh

**Affiliations:** College of Pharmacy and Research Institute of Pharmaceutical Sciences, Seoul, South Korea

**Keywords:** drug repositioning, castration resistant prostate cancer, gene expression, drug activity, synergic effect

## Abstract

**Purpose:** Most prostate cancers (PCs) initially respond to androgen deprivation therapy (ADT), but eventually many PC patients develop castration resistant PC (CRPC). Currently, available drugs that have been approved for the treatment of CRPC patients are limited. Computational drug repositioning methods using public databases represent a promising and efficient tool for discovering new uses for existing drugs. The purpose of the present study is to predict drug candidates that can treat CRPC using a computational method that integrates publicly available gene expression data of tumors from CRPC patients, drug-induced gene expression data and drug response activity data.

**Methods:** Gene expression data from tumoral and normal or benign prostate tissue samples in CRPC patients were downloaded from the Gene Expression Omnibus (GEO) and differentially expressed genes (DEGs) in CRPC were determined with a meta-signature analysis by a metaDE R package. Additionally, drug activity data were downloaded from the ChEMBL database. Furthermore, the drug-induced gene expression data were downloaded from the LINCS database. The reversal relationship between the CRPC and drug gene expression signatures as the Reverse Gene Expression Scores (RGES) were computed. Drug candidates to treat CRPC were predicted using summarized scores (sRGES). Additionally, synergic effects of drug combinations were predicted with a Target Inhibition interaction using the Minimization and Maximization Averaging (TIMMA) algorithm.

**Results:** The drug candidates of sorafenib, olaparib, elesclomol, tanespimycin, and ponatinib were predicted to be active for the treatment of CRPC. Meanwhile, CRPC-related genes, in this case *MYL9, E2F2, APOE*, and *ZFP36*, were identified as having gene expression data that can be reversed by these drugs. Additionally, lenalidomide in combination with pazopanib was predicted to be most potent for CRPC.

**Conclusion:** These findings support the use of a computational reversal gene expression approach to identify new drug and drug combination candidates that can be used to treat CRPC.

## Introduction

Drug repurposing or repositioning is a strategy for identifying new indications for approved or investigational drugs that are outside the scope of the original medical indication (1). This strategy offers an advantage in that the cost of bringing a repurposed drug to market has been estimated to be US$300 million on average, compared to estimates of approximately $2–3 billion for a new drug (2).

Drug–disease similarity approaches aim to identify shared therapeutic applications for drugs (3) while drug–drug similarity approaches aim to identify shared mechanisms of action for drugs (4). Recently, interest in the use of genomics-based drug repositioning to aid and accelerate the drug discovery process has increased (5). Drug development strategies based on gene expression levels are advantageous in that they do not require a large amount of a priori knowledge pertaining to particular diseases or drugs (6, 7). Large public datasets such as the Gene Expression Omnibus (GEO) at the National Center for Biotechnology Information (NCBI) (8), the Cancer Cell Line Encyclopedia (CCLE) (9), and the Library of Integrated Network-Based Cellular Signatures (LINCS) (10, 11), describing chemical and biological disease entities or gene expression data and the relationships between them, provide an efficient approach by which to reposition existing drugs for new indications (5, 12). Recently, the reverse gene expression scores (RGES) computation method was developed as a one of the powerful drug repositioning tools to predict drug candidates (13). The RGES computation method was applied to find drug candidates for CRPC in this study.

Prostate cancer (PC) was the cancer with the highest incidence worldwide and the leading cause of cancer deaths for men in 2015 (14). Although PC mortality in Western countries has declined due to early diagnosis and treatment, incidence rates of PC continue to increase in the developing countries (15). Androgens and androgen receptors (ARs) may play key roles in the initiation and progression of PC (16). As Huggins and Hodges discovered that androgen-deprivation therapy (ADT) with surgical castration to reduce testicular testosterone could suppress PC progression (17), ADT has been the standard therapy to treat PC (16, 18).

Different therapeutic approaches to target androgen and AR signals after surgical or chemical castration were developed by combining ADT with various anti-androgens, including the steroidal anti-androgens cyproterone acetate (19) and megestrol acetate (19), and non-steroidal anti-androgens, including nilutamide (20), flutamide (21), and bicalutamide (22). Most PCs initially shrink in response to ADT, but eventually most types of ADT with anti-androgens fails and PC patients develop castration resistant PC (CRPC) (16). When this occurs, chemotherapeutic approaches such as docetaxel may be considered (23). Although recently enzalutamide, apalutamide, and abiraterone acetate were approved for the treatment of CRPC (24, 25), available drugs that have been approved for the treatment of CRPC patients are limited (26). Therefore, repositioning for CRPC is challenging and numerous preclinical or clinical trial studies deriving from repositioning approaches, including those focusing on itraconazole (27), phentolamine (28), and niclosamide (29), have been continually conducted.

Primary PC has relatively few genomic aberrations compared to other cancers (30). However, detailed spatial sampling and sequencing of prostate tumors has identified significant heterogeneity within multifocal tumors in the same patient (31, 32). In the case of CRPC, it has been shown to remain dependent on the AR signaling pathway by various mechanisms even during the systemic depletion of androgens (33).

The purpose of this study is to predict drug candidates that can treat CRPC using a computational method that integrates publicly available gene expression data of tumors in CRPC patients, drug-induced gene expression data and drug response activity data.

## Materials and Methods

### Collection of Gene Expression Data

Publicly available gene expression data for CRPC related studies were identified in the GEO database hosted by the NCBI (http://www.ncbi.nlm.nih.gov/geo). A search of the GEO database was conducted in July of 2018 using “prostate cancer,” “castration,” “resistance,” and “refractory” as a key search phrases. The results were filtered using the search terms *Homo sapiens*, expression profiling by array, and expression profiling by high throughput sequencing. Only original experimental datasets that compared the expression levels of mRNAs between CRPC tumor and normal or benign tissues were selected in CRPC patients. Additionally, gene expression data of human prostate adenocarcinoma cell lines were downloaded from CCLE (version 2.7. updated 2015 https://portals.broadinstitute.org/ccle) (9).

### Preprocessing of Gene Expression Data

The GEO accession number, Gene Expression Omnibus platforms (GPL) access number, number of cases and controls, sample type, and gene expression data were extracted from each of the identified datasets. Gene expression data were individually log2-based transformed and normalized. If there were multiple probes for the same gene, the probe with the highest interquartile range was selected for that gene expression level. All probe sets on different platforms were re-annotated to use the most recent NCBI Entrez Gene Identifiers (Gene IDs), and the Gene IDs were used to cross-map genes among the different platforms. Only genes present in all selected platforms were considered. The R packages MetaQC (34) was used for quality control (QC) of gene expression data. The mean rank of all QC measures in each dataset was also determined as a quantitative summary score by calculating the ranks of each QC measure among all included datasets.

### Identification of Disease Gene Expression Signatures

The R package MetaDE (35–38) was used to identify the DEGs in meta-analysis of CRPC. A moderated *t*-statistic was used to calculate *P*-values for each dataset, and a meta-analysis was conducted with a fixed effect model (39). Additionally, the degree of similarities among the gene expression data between CRPC tumor samples from the GEO and PC cell lines from the CCLE were assessed.

### Identification of Compound Gene Expression Signatures

The L1000 landmark transcript values (Level 4) of 978 landmark genes from LINCS as of May of 2018 were downloaded from LINCS cloud storage (http://lincscloud.org/) hosted by the Broad Institute (40). Cell lines described in LINCS, CCLE, and ChEMBL (version 24 1st Sep 2017, https://www.ebi.ac.uk/chembl/) (41) were mapped using PC cell line names followed by manual inspection. Meta-information for compound-induced gene expression values, in this case cell line types as well as treatment durations and drug concentrations, was retrieved. Only L1000 signatures with the annotation “is gold,” indicating the highest quality of aggregate data were used for further analyses.

### Collection of Compound Activity Data

Compound activity data, described as the half maximal inhibitory concentrations (IC_50_) in PC cell lines were retrieved from ChEMBL. As the IC_50_ values for a given compound could vary for the same cell line across different studies, the median IC_50_ value was used. Compounds included in the ChEMBL and LINCS were manually mapped using International Union of Pure and Applied Chemistry International Chemical Identifier keys. Additionally, the area-under-the-curve (AUC) values for compound activity data in the PC cell lines were retrieved from the Cancer Therapeutic Response Portal (CTRP ver 2, https://portals.broadinstitute.org/ctrp.v2.1/) (42). Sensitivity levels were measured in the form of cellular ATP levels as a surrogate for cell number and growth using CellTiter-Glo assays (43). A compound-performance score was computed at each concentration of compound. Median AUC values across various cell lines were used. Compounds were categorized into active (IC_50_ <10 μM) and inactive groups (IC_50_≥10 μM) based on their activities in cell lines. An IC_50_ value of 10 μM was chosen as an activity threshold given that compounds with IC_50_ ≥10 μM in primary screenings are often not pursued (44).

### Computation and Summarization of RGES

The method used to calculate the reverse gene expression score (RGES) was adapted from the previously described Connectivity Map method (45) and RGES computational method (13). Briefly, genes were initially ranked according to their expression levels for each compound. An enrichment score for each set of up- and down-regulated genes in CRPC was computed based on their positions in the ranked list. RGES values emphasize the reversal correlation by capturing the reversal relationship between the DEGs and compound-induced changes in the gene expression levels. Therefore, a lower negative RGES indicates a higher likelihood of reversing changes in the gene expression of CRPC, and *vice versa*. In addition, the Spearman's correlation coefficient, the Pearson correlation coefficient, and the cosine similarity were computed between DEGs in CRPC and compound activities, as an alternate method for computing the reversal relationship between DEGs and the active compounds (46). The databases used can list multiple gene expression levels associated with one compound due to testing involving different cell lines, treatment concentrations and durations of compounds. This resulted in multiple RGESs for one compound that could reverse disease gene expression. Given these variations, summarized RGESs (sRGES) were weighted and calculated. Results obtained for a 10 μM drug concentration and a 24 h treatment time were used to define the reference conditions.

### Identification of Reversed Genes

In cases for which multiple gene expression values yielded multiple RGES values for one compound, a median RGES value was calculated from the PC cell lines. In cases for which multiple compound activity IC_50_ data were available for one compound, median IC_50_ values were calculated. Each gene expression datum was sorted by its expression value. Upregulated genes were ranked highly (i.e., on the top), whereas downregulated genes were assigned a low rank (i.e., on the bottom). Among the upregulated genes, reversal genes were defined as those that were ranked lower in the active group (IC_50_ <10 μM) than in the inactive group (IC_50_ ≥ 10 μM). In contrast, among the downregulated genes, the reversal genes were defined as those that were ranked higher in the active group than the inactive group. A leave-one-compound-out cross-validation approach was used to find genes having reversed expression (47). For each trial, one compound was removed and the reversed genes were then identified using the approach described above. Only those genes that were significantly reversed in all trials were retained. The genes with adjusted *P* < 0.25 in all trials were considered as reversal genes.

### Prediction of Synergic Effects

To predict the synergic effects of drug combinations based on the interactions between drugs and the identified targets, RGES values was used for Target Inhibition Inference using the Maximization and Minimization Averaging (TIMMA) algorithm (48). The synergic scores were calculated with the TIMMA-R package (49). The synergic addictive score was defined as *S*_*a*_(*i, j*) = *y*(*i, j*) − (*y*(*i*) + *y*(*j*)) and the synergic multiplicative score was defined as *S*_*m*_(*i, j*) = *y*(*i, j*) − (*y*(*i*) × *y*(*j*)). The synergic highest agent score was defined as *S*_*l*_(*i, j*) = *y*(*i, j*) − max(*y*(*i*), *y*(*j*)). An average synergy score was defined as $S\left(d1,\;d2\right)=\frac{1}{n}\;\underset{i\in d1,\;j\in d2}{\sum }S\left(i,j\right)$. The predicted sensitivity was defined as *Sensitivity* = *expectation* + *synergy*.

### Statistical Analysis

The degree of similarity in gene expressions between CRPC tumor samples from the GEO and PC cell lines from the CCLE were assessed by a Spearman's rank correlation test, as were these similarity degrees between RGES and IC_50_ values from ChEMBL or AUC values from CCLE. A Wilcoxon signed-rank test was used to assess differences between RGES across active (IC_50_ <10 μM) and inactive compounds (IC_50_ ≥ 10 μM), the same and different cell lines, higher (≥10 μM) and lower (<10 μM) drug concentrations, and longer (≥24 h) and shorter (<24 h) treatment durations. *P*-values were adjusted with the Benjamini and Hochberg's false discovery rate method to correct for multiple testing.

## Results

### Inclusion of CRPC Gene Expression Datasets

**Figure 1:**
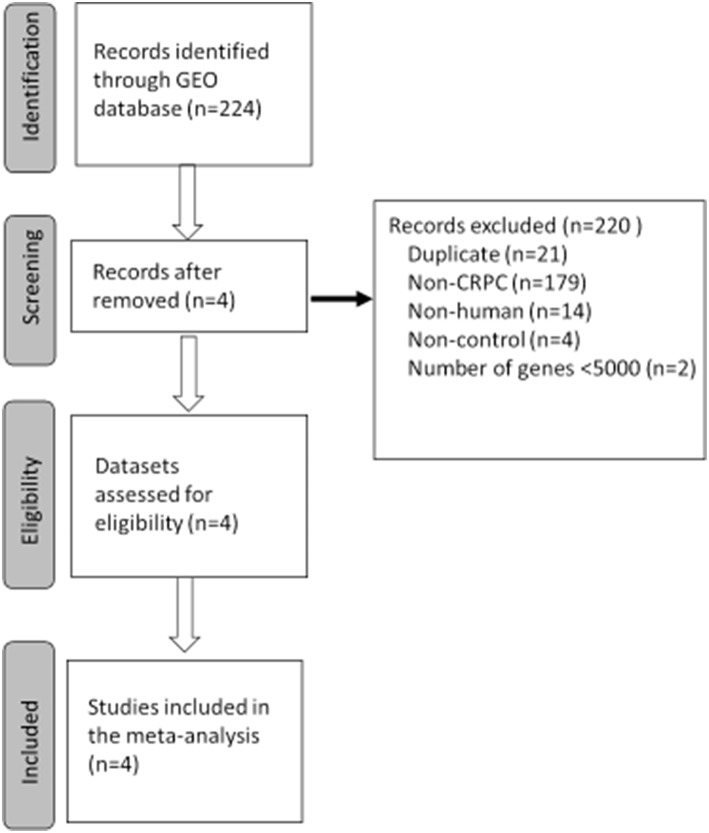
Flowchart of the selected process to select gene expression datasets for meta-analysis of castration resistant prostate cancer (CRPC). GEO, Gene Expression Omnibus.

The study selection process for finding CRPC disease signatures is outlined in Figure 1. A total of 224 GEO Series Experiments (GSEs) were searched. A number of GSEs were excluded due to duplicated data (*n* = 21), disease (*n* = 179), non-human (*n* = 14), non-control (neither normal nor benign tissues) (*n* = 4), and number of genes <5,000 (*n* = 2). Finally, the four datasets of GSE3325, GSE35988, GSE70768, and GSE80609 were selected for further analysis after a MetaQC analysis (Supplementary Table S1). Detailed information about the downloaded CRPC gene expression datasets is summarized in Supplementary Table S2. GSE35988 contained gene expression data from the GPL6480 and GPL6848 platforms. Tumor gene expression signatures in CRPC were analyzed for 178 samples by comparing RNA expression data for 64 tumors and 114 normal or benign tissues from those four datasets.

### Gene Expression Signatures of CRPC

The workflow for the exploration of the compounds using the calculated RGES values is presented in Supplementary Figure S1. Corresponding probes on each platform were re-annotated with the most recent NCBI Entrez Gene IDs and then mapped to yield 7,825 unique common genes across the five different platforms. A fixed-effect model method was used by combining the *P*-values using the MetaDE package. Among the gene expression signatures, 53 genes showed increased expression levels in tumors compared to normal or benign tissues (adjusted *P* < 0.001, log_2_foldchange > 1.5), whereas 42 genes showed decreased expression levels in tumors compared to normal or benign tissues (adjusted *P* < 0.001, log_2_foldchange < –1.5; Supplementary Table S3).

### Similarity in Gene Expressions Between Tumor Samples and PC Cell Lines

The degree of similarity in the gene expression levels between CRPC tumor samples from the GEO and PC cell lines from the CCLE was assessed by a ranked Spearman's correlation test. Gene expression data for eight PC cell lines were included in the CCLE (Supplementary Table S4). The top 5,000 genes in these cell lines were ranked according to their interquartile range across all PC cell lines used. Among them, <0.1% of genes had expression levels in tumor samples from the GEO that did not correlate with those in these cell lines.

### Computation of RGES Values

**Figure 2:**
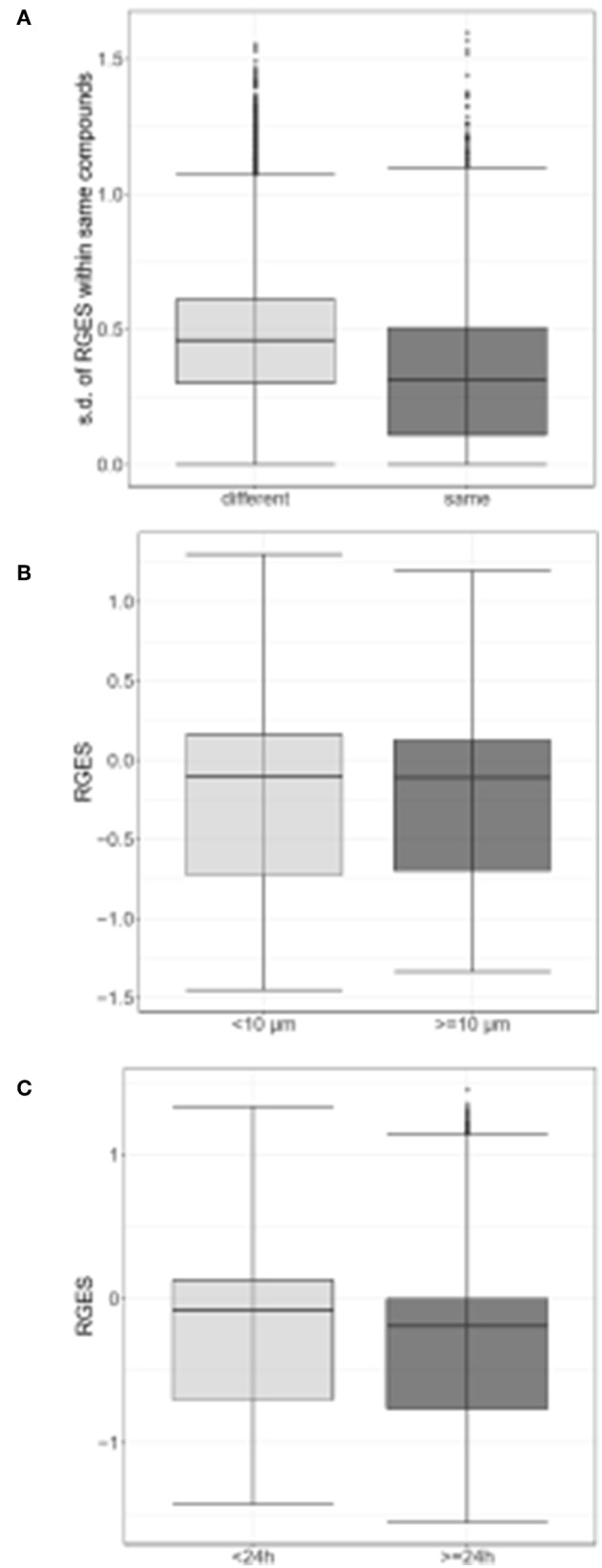
Differences in reverse gene expression scores (RGES) under the various biological conditions. **(A)** Standard deviation (s.d.) of reverse gene expression scores (RGES) of individual compounds across different cell lines (gray) vs. across replicates within the same cell line (black gray). **(B)** RGES distribution between drug concentrations<10 μM (gray) and ≥10 μM (black gray). **(C)** RGES distribution between treatment durations<24 h (gray) and ≥24 h (black gray). Treatment duration and compound concentration were categorized based on compound data in LINCS. *P*-value was calculated using a Wilcoxon signed-rank test.

**Figure 3:**
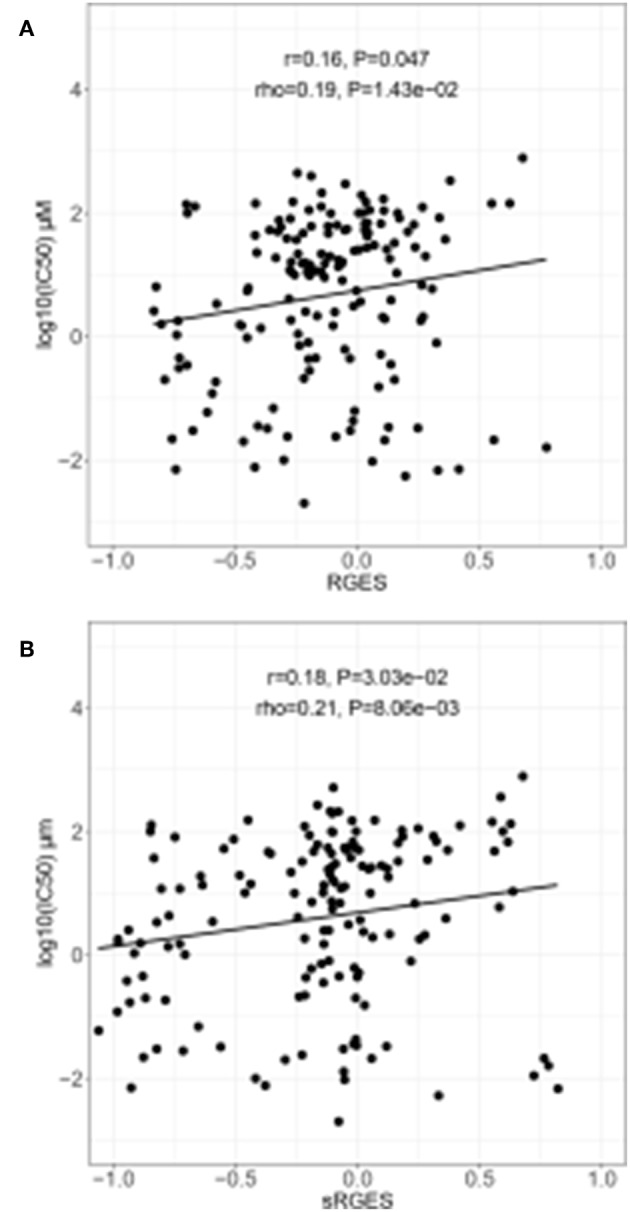
Correlation between drug efficacy and reverse gene expression score (RGES) in PC3 cancer cell lines. **(A)** Correlation between RGES and drug efficacy (IC_50_) by linear regression and Spearman's correlation tests. **(B)** Correlation between drug efficacy (IC_50_) and summarized reverse gene expression score (sRGES) for all cancer cell lines using a linear regression and a Spearman's correlation tests. IC_50_, half maximal inhibitory concentration.

Changed expression values of 978 landmark genes after a compound treatment of human prostate adenocarcinoma PC3 cell lines with 172 compounds in the LINCS data as drug signatures were used for the computation of the RGES values. The median IC_50s_ values for 12,895 compounds used to treat PC cancer cell lines listed in the ChEMBL were used for the RGES computation. The changed expression values of 95 DEGs after extraction from the set of LINCS landmark genes as disease signatures were also used for computation. Variations in the RGES values were evaluated under various biological conditions. The RGES values showed larger variations across different cell lines relative to those within different replicates of the same cell line when the same concentration and treatment duration for a compound were used (*P* < 2.2 × 10^−16^; Figure 2A). In addition, higher compound concentrations (>10 μM) had lower RGES values than lower concentrations (<10 μM, *P* = 1.46 × 10^−4^; Figure 2B) when a compound was tested on the same cell line at the same concentration. Likewise, longer treatment durations (≥24 h) were associated with lower RGES values compared to shorter durations (<24 h) (*P* < 2.2 × 10^−16^; Figure 2C). The RGES values for the compounds were evaluated by examining the correlations with their activities in the same cell line. Finally, the RGES values were correlated with the IC_50_ values for the compounds (Spearman correlation rho = 0.19, *P* = 1.43 × 10^−2^; Figure 3A).

### Summarization and Evaluation of RGES Outcomes

**Figure 4:**
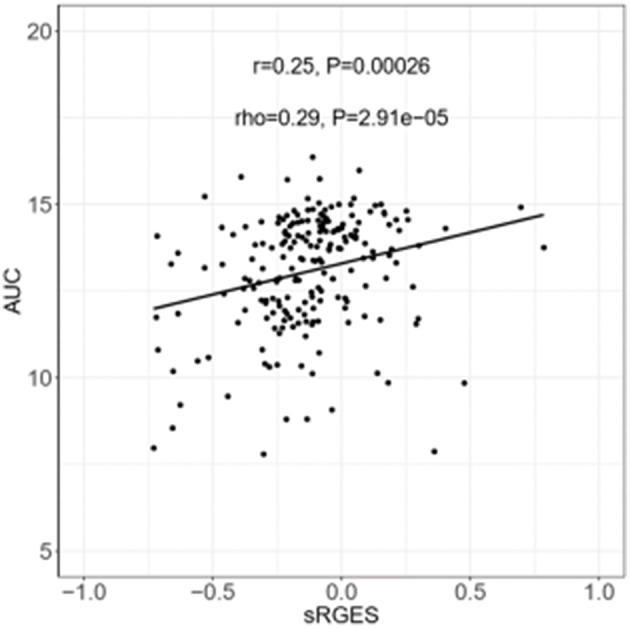
Correlation between AUC and sRGES of compounds. AUC data were retrieved from CTRP. Sensitivity levels were measured in the form of cellular ATP levels as a surrogate for cell number and growth using CellTiter-Glo assays. A compound-performance score was computed at each concentration of compound. Median values were used to summarize AUC across all prostate cancer cell lines examined. A Spearman's correlation test was used to analyze the correlation between sRGES and AUC. AUC, areas under the concentration-response curve. CTRP, the cancer therapeutic response portal.

sRGES values were computed by weighting various cell lines, compound concentrations, and treatment durations. A number of known methods were used to summarize the RGES outcomes and obtain sRGES values (Supplementary Table S5). The calculated sRGES values for each compound were significantly correlated with drug activity levels (Spearman correlation rho = 0.21 and *P* = 8.06 × 10^−3^; Figure 3B). Additionally, CTRP was used as an external data set to confirm the correlation between the reversal potency and the compound activity. Compound activity data expressed as AUC values for 558 compounds tested in PC cell lines were collected from CTRP. After the sRGES computations, the median AUC values across multiple cell lines were used to evaluate the sRGES outcomes. The sRGES values were significantly correlated with the AUC values (rho = 0.29, *P* = 2.91 × 10^−5^; Figure 4).

### Identification of Reversed Genes and Predictions of Compounds

**Figure 5:**
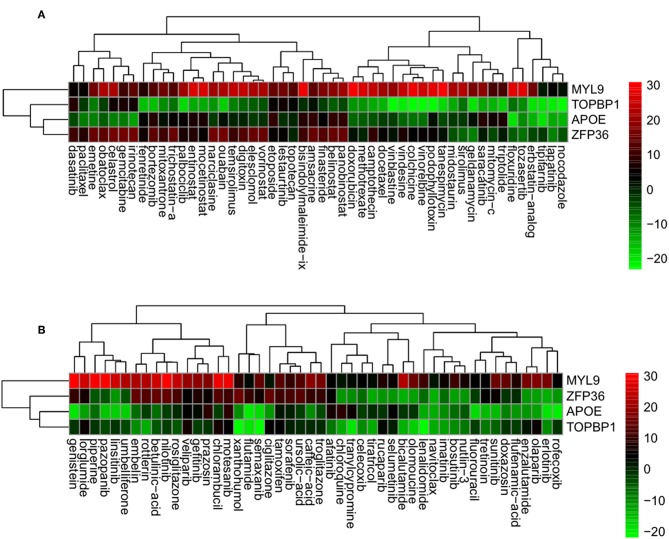
Genes having reversed expression in response to treatment with active **(A)** and inactive **(B)** compounds. Low rank and high rank suggests that the gene expression is down- and upregulated, respectively, by the corresponding compound. The heatmap indicates the relative position of a gene in ranked drug expression data. Position are normalized and compound columns are ordered according to IC_50_. Red and green colors indicate up- and down-regulation, respectively, after compound treatment.

Using the correlation between the sRGES values and the compound activity, compounds having high reversal potency for PC were identified. Next, genes having expression levels that were reversed by the active compounds were predicted by a leave-one-compound-out approach. The four genes that showed significant reversal of expression following treatment with PC cell lines with the active compounds included the following: (i) myosin light chain 9 (*MYL9*), (ii) DNA topoisomerase 2-binding protein 1 (*TOPBP1*), (iii) Apolipoprotein E (*APOE*), and (iv) zinc finger protein 36 (*ZFP36*) (Figures 5A,B). Fifty compounds were determined to be active compounds against CRPC (Figure 5A), while 48 compounds were determined to be inactive compounds (Figure 5B, Supplementary Table S6). The active drugs against CRPC identified by our analysis contained the tyrosine kinase inhibitors dasatinib, lapatinib, lestaurtinib, and saracatinib; the histone acetylation inhibitors belinostat, entinostat, mocetinostat, panobinostat, trichostatin-A, and vorinostat and the heat shock inhibitors elesclomol and geldanamycin.

### Prediction of the Synergic Effect

**Figure 6:**
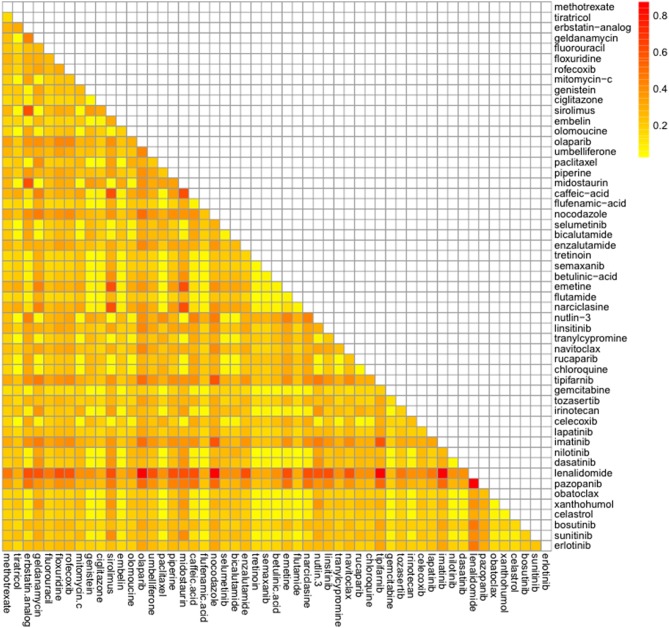
Predicted synergic drug combinations against castration resistant prostate cancer. Predicted sensitivity scores were calculated by the Target Inhibition Interaction using Maximization and Minimization Averaging algorithm. The bar on the right shows predicted synergic scores.

Combination of 98 drug candidates were employed the TIMMA. Of all these drug combinations, the highest synergy sensitivity score was predicted for the combination of lenalidomide and pazopanib (Figure 6, Supplementary Table S7). Next, the synergic sensitivity score orders were lenalidomide combined with olapanib, nocodazole, tipifarnib, and imatinib and their corresponding targets were *APOE, ZFP36, E2F2*, and *MYL9*.

## Discussion

The computational approaches used in systemic analyses of large amounts of data such as gene expression values, genotypes, and chemical structure similarities for predictive repositioning offer a relatively quick and mechanistic method of identifying new application of existing drugs that may be translated into clinical applications (50). In this study, computational methods using public database were used for the purpose of identifying drug repositioning candidates for treatment of CRPC. Several drug candidates were identified, as well as DEGs in CRPC whose expression can be reversed by these agents. We used public cancer genomic and pharmacologic databases to demonstrate the reversal potency relationship between DEGs and drug activities, and to predict potential new drug candidates for CRPC.

Our results showed that the ability of drugs to reverse DEGs was correlated with drug activity in CRPC, although this correlation was highly dependent on the cell lines as well as the treatment concentration and duration of the drugs. The positive correlation between sRGES and IC_50_ values indicated that combining disease gene expression data derived from clinical samples with drug gene expression data obtained from results with *in vitro* cell lines could be used to predict drug activities.

In our study, four genes, *MYL9, E2F2, APOE*, and *ZFP36*, showed reversed expression in response to 50 active compounds in CRPC. To the best of our knowledge, this is the first study focusing on drug repositioning using a computational reversal gene expression approach in relation to CRPC. *MYL9* (myosin light chain 9) has been reported to play an important role in tumor progression in PC (51). Loss of the RB function facilitates the development of CRPC via E2F-mediated upregulation of the AR (52). *ZFP36* is reportedly involved in the progression and prognosis of PC (53). As cholesterol is known to be a potential target for CRPC, *ApoE* has been suggested to play a potential role in prostate cancer progression (54). These genes showed reversed expression levels and thus may be feasible as therapeutic targets for CRPC.

Among the active drugs against CRPC identified by our analysis, the histone deacetylase inhibitors, vorinostat (55), and panobinostat (56), the tyrosine kinase inhibitors, dasatinib (57) and lapatinib (58), and a poly (ADP-ribose) polymerase (PARP) inhibitor, olaparib (59), have gone through phase I and phase II clinical trials for CRPC patients. Additionally, the docetaxel FDA-approved for metastatic CRPC (60) was identified as an active drug in our results. Bicalutamide and flutamide used as a hormonal therapy against CRPC have low cytotoxic activities, resulting that they have been identified as inactive drugs in our study. The cytotoxic chemotherapy is most effective when given in combination to achieve an additive or synergistic effect and a targeted therapy involving more than one drug increases powerful antitumor effects and overcomes resistance mechanisms (61). A combination of high-throughput screening of all licensed drugs has been carried out in an attempt to discover synergistic interactions (62). Therefore, the synergic effects of the candidate drugs identified were examined in our study. The immunomodulatory, drug lenalidomide combined with a tyrosine kinase inhibitor, pazopanib was most potent against CRPC in our result. Several clinical trials of lenalidomide- or pazopanib-based regimens for CRPC were conducted (63–65). The phase I/II study of lenalidomide in combination with sunitinib was conducted in patients with advanced or metastatic renal cell carcinoma (66). Despite the fact that the combination of pazopanib and lenalidomide has been reported to induce synergistic cytotoxicity in multiple myeloma (67), this drug combination has not yet been tested yet in CRPC cases.

After CRPC development, PC switches from an endocrine-driven disease to a paracrine- or autocrine-driven disease. Therefore, CRPC represents an increased opportunity to accumulate genomic aberrations and is riddled with aggressive and heterogeneous clones. Strengths of this work is that it leverages publically available datasets to identify candidates for drug repositioning targets and theoretically, this could be a much cheaper and faster way to identify promising new leads. A limitation of this study is that the CRPC disease gene expression datasets from the GEO are not uniformly associated with clinical outcomes or CRPC etiologies. CRPC is a heterogeneous disease that might be driven by different pathways, depending on prior treatments and this approach essentially treats CRPC as a single entity. The drug efficacy of the predicted compounds may also vary because the CRPC tissue states varied for individual patients. Therapeutic efficacy is more complex than a simple correlation of gene expression levels with drugs and diseases. Therefore, our findings for drug candidates will require further preclinical testing and demonstration in clinical trials. As performing randomized trials in relation to a rare cancer disease is challenging, a computational drug repositioning approach with public gene expression databases may become a quite useful strategy for treating rare types of cancer.

In summary, our computational approach combined disease gene expression with drug-induced expression data in CRPC to identify new drugs and target genes as CRPC therapies. This approach, can also be used to predict the efficacy of new drug candidates to treat CRPC. This computational approach could be broadly applied to other rare forms of cancer for which reliable gene expression data are available.

## Author Contributions

I-WK and JO contributed conception and design of the study. I-WK organized the database and wrote the first draft of the manuscript. I-WK and JK contributed to data collection and performed the statistical analysis. All authors contributed to manuscript revision, read, and approved the submitted version.

### Conflict of Interest Statement

The authors declare that the research was conducted in the absence of any commercial or financial relationships that could be construed as a potential conflict of interest.
