# Supplementary material for: Screening of Drug Repositioning Candidates for Castration Resistant Prostate Cancer
Source: Front Oncol. 2019 Jul 23;9:661. doi: 10.3389/fonc.2019.00661 (PMC6664029; doi:10.3389/fonc.2019.00661)
Supplement: Supplementary file 1 [file Data_Sheet_1.docx]

Supplementary Material


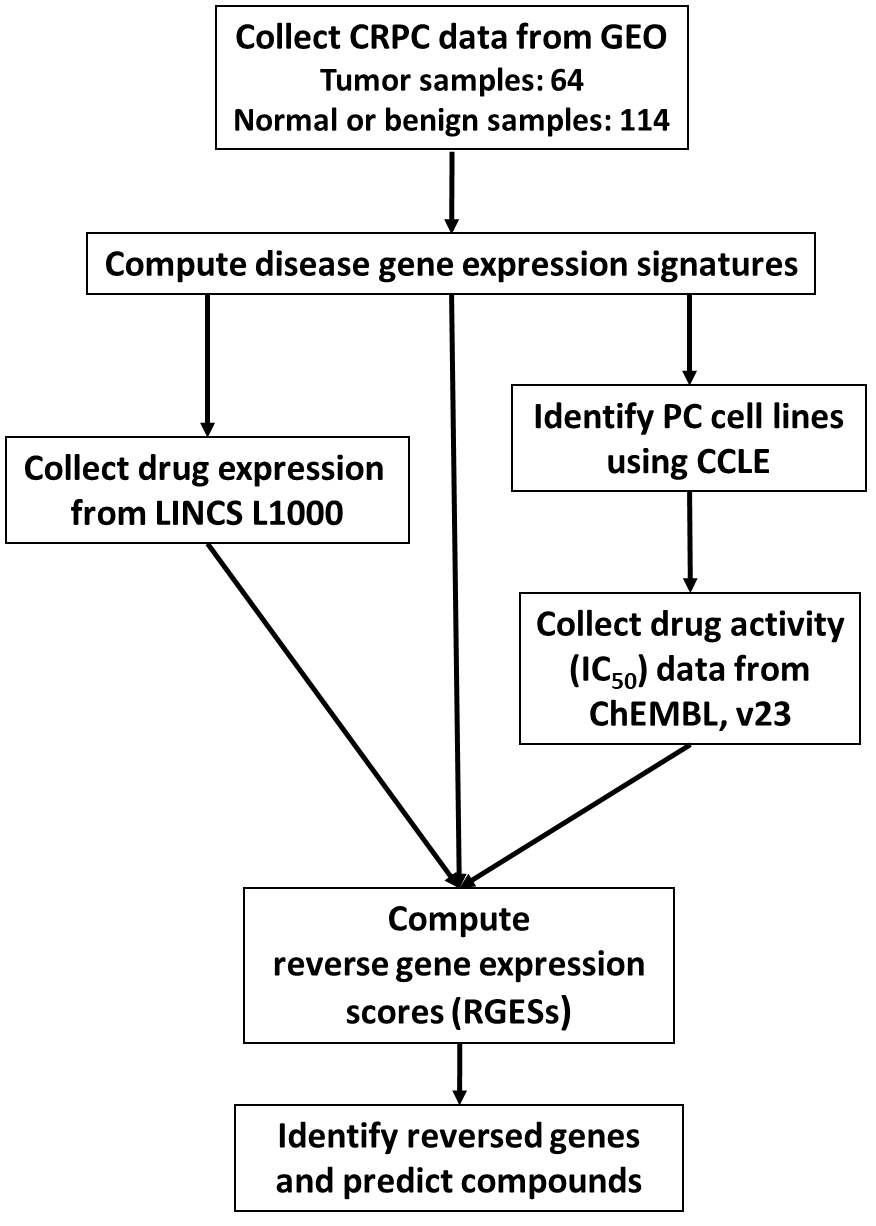


**Supplementary Figure S1**. Workflow to determine the reverse gene expression score (RGES) using disease and drug gene expression data. The public database GEO was used to create cancer gene expression signatures; LINCS L1000 was used as the drug signature database; ChEMBL was used as the drug efficacy database; and CCLE was used to map cell lines among databases. GEO, Gene Expression Omnibus; CRPC, castration resistant prostate cancer; LINCS, The Library of Integrated Network-Based Cellular Signatures; ChEMBL, a chemical database of bioactive molecules maintained by the European Bioinformatics Institute of the European Molecular Biology Laboratory; CCLE, Cancer Cell Line Encyclopedia.

**Supplementary Table S1**. The quality control results using MetaQC

| GSE ID | IQC | EQC | CQCg | CQCp | AQCg | AQCp | Rank |
| --- | --- | --- | --- | --- | --- | --- | --- |
| GSE35988_GPL6480 | 4.91 | 2.46 | 53.17 | 172.31 | 14.34 | 85.76 | 1.83 |
| GSE3325 | 1.56* | 3.40 | 30.40 | 103.79 | 6.98 | 79.57 | 2.33 |
| GSE35988_GPL6848 | 5.31 | 3.35 | 11.42 | 65.74 | 0.32 | 25.89 | 3.17 |
| GSE70768 | 4.50 | 3.22 | 16.43 | 24.38 | 3.58 | 10.87 | 3.83 |
| GSE80609 | 2.04 | 3.26 | 8.37 | 40.14 | 0.84* | 44.52 | 3.83 |

GSE, Gene Expression Omnibus Series Experiments; GPL, Gene Expression Omnibus platforms; IQC, Internal quality control; EQC, External quality control; CQCg, Consistency quality control in genes; CQCp, Consistency quality control in pathways; AQCg, Accuracy quality control in genes; AQCp, Accuracy quality control in pathways; Rank, a standardized mean rank; **P* value not signiﬁcant after a Bonferroni correction

**Supplementary Table S2.** Information of the gene expression datasets from the GEO

| GSE ID | GPL ID | Platform | Number of Sample  (tumor/normal or benign) |
| --- | --- | --- | --- |
| GSE3325 | GPL570 | Affymetrix GeneChip Human Genome U133 Plus 2.0 Array | 4/4 |
| GSE35988 | GPL6480 | Agilent-014850 Whole Human Genome Microarray 4x44K G4112F | 27/12 |
|  | GPL6848 | Agilent-012391 Whole Human Genome Oligo Microarray G4112A | 8/16 |
| GSE70768 | GPL10558 | Illumina HumanHT-12 V4.0 Beadchip | 13/74 |
| GSE80609 | GPL11154 | Illumina HiSeq 2000 sequencing system | 12/8 |

GSE, Gene Expression Omnibus Series Experiments; GPL, Gene Expression Omnibus platforms

**Supplementary Table S3**. Disease gene expression signatures for castration resistant prostate cancer

| GeneID | symbol | baseMean | log2FoldChange | *P* value  (×10^-20^) | Adjusted *P* value  (×10^-20^) |
| --- | --- | --- | --- | --- | --- |
| 55 | *ACPP* | 0.24 | -2.05 | 1.00 | 6.61 |
| 203054 | *ADCK5* | 2.86 | 1.52 | 1.00 | 6.61 |
| 128 | *ADH5* | 0.29 | -1.78 | 1.00 | 6.61 |
| 10550 | *ARL6IP5* | 0.32 | -1.64 | 1.00 | 6.61 |
| 51309 | *ARMCX1* | 0.27 | -1.87 | 1.00 | 6.61 |
| 563 | *AZGP1* | 0.30 | -1.74 | 1.00 | 6.61 |
| 140707 | *BRI3BP* | 3.01 | 1.59 | 1.00 | 6.61 |
| 28970 | *C11ORF54* | 0.35 | -1.51 | 1.00 | 6.61 |
| 285382 | *C3ORF70* | 0.29 | -1.77 | 1.00 | 6.61 |
| 801 | *CALM1* | 0.35 | -1.52 | 1.00 | 6.61 |
| 890 | *CCNA2* | 1.78 | 1.51 | 1.00 | 6.61 |
| 991 | *CDC20* | 1.56 | 1.66 | 1.00 | 6.61 |
| 10602 | *CDC42EP3* | -2.56 | -1.82 | 1.00 | 6.61 |
| 148170 | *CDC42EP5* | -2.67 | -1.57 | 1.00 | 6.61 |
| 83461 | *CDCA3* | 1.92 | 1.66 | 1.00 | 6.61 |
| 113130 | *CDCA5* | 2.25 | 2.02 | 1.00 | 6.61 |
| 30850 | *CDR2L* | 1.55 | 1.84 | 1.00 | 6.61 |
| 81620 | *CDT1* | 1.45 | 1.51 | 1.00 | 6.61 |
| 1951 | *CELSR3* | 1.87 | 2.03 | 1.00 | 6.61 |
| 1063 | *CENPF* | 1.97 | 1.97 | 1.00 | 6.61 |
| 63922 | *CHTF18* | 1.85 | 2.17 | 1.00 | 6.61 |
| 1264 | *CNN1* | -4.05 | -1.82 | 1.00 | 6.61 |
| 1346 | *COX7A1* | -1.85 | -1.55 | 1.00 | 6.61 |
| 1490 | *CTGF* | -2.05 | -1.76 | 1.00 | 6.61 |
| 9265 | *CYTH3* | 1.33 | 1.55 | 1.00 | 6.61 |
| 1674 | *DES* | -2.79 | -1.76 | 1.00 | 6.61 |
| 1843 | *DUSP1* | -1.60 | -1.65 | 1.00 | 6.61 |
| 1870 | *E2F2* | 2.04 | 1.90 | 1.00 | 6.61 |
| 2101 | *ESRRA* | 1.59 | 1.60 | 1.00 | 6.61 |
| 2297 | *FOXD1* | 2.27 | 1.71 | 1.00 | 6.61 |
| 152007 | *GLIPR2* | -1.76 | -1.83 | 1.00 | 6.61 |
| 221914 | *GPC2* | 1.62 | 1.70 | 1.00 | 6.61 |
| 23171 | *GPD1L* | -2.06 | -1.69 | 1.00 | 6.61 |
| 84264 | *HAGHL* | 2.03 | 2.12 | 1.00 | 6.61 |
| 3779 | *KCNMB1* | -2.65 | -1.62 | 1.00 | 6.61 |
| 253980 | *KCTD13* | 1.95 | 1.65 | 1.00 | 6.61 |
| 54793 | *KCTD9* | -1.47 | -1.60 | 1.00 | 6.61 |
| 10112 | *KIF20A* | 2.33 | 1.58 | 1.00 | 6.61 |
| 90990 | *KIFC2* | 1.60 | 1.86 | 1.00 | 6.61 |
| 54923 | *LIME1* | 1.32 | 1.54 | 1.00 | 6.61 |
| 84823 | *LMNB2* | 1.45 | 1.65 | 1.00 | 6.61 |
| 158056 | *MAMDC4* | 1.33 | 1.60 | 1.00 | 6.61 |
| 92312 | *MEX3A* | 1.55 | 1.55 | 1.00 | 6.61 |
| 4236 | *MFAP1* | -1.58 | -1.66 | 1.00 | 6.61 |
| 4320 | *MMP11* | 1.32 | 1.51 | 1.00 | 6.61 |
| 4494 | *MT1F* | -1.52 | -1.51 | 1.00 | 6.61 |
| 4637 | *MYL6* | -1.59 | -1.52 | 1.00 | 6.61 |
| 10398 | *MYL9* | -2.56 | -1.85 | 1.00 | 6.61 |
| 4744 | *NEFH* | -3.01 | -2.62 | 1.00 | 6.61 |
| 126382 | *NR2C2AP* | 1.56 | 1.67 | 1.00 | 6.61 |
| 11339 | *OIP5* | 1.42 | 1.56 | 1.00 | 6.61 |
| 55003 | *PAK1IP1* | -2.23 | -2.38 | 1.00 | 6.61 |
| 25849 | *PARM1* | -2.48 | -2.24 | 1.00 | 6.61 |
| 55742 | *PARVA* | -3.16 | -1.60 | 1.00 | 6.61 |
| 5121 | *PCP4* | -2.83 | -2.01 | 1.00 | 6.61 |
| 64714 | *PDIA2* | 1.91 | 1.57 | 1.00 | 6.61 |
| 8985 | *PLOD3* | 1.68 | 1.52 | 1.00 | 6.61 |
| 5437 | *POLR2H* | 2.17 | 1.72 | 1.00 | 6.61 |
| 26472 | *PPP1R14B* | 2.74 | 2.52 | 1.00 | 6.61 |
| 84988 | *PPP1R16A* | 2.08 | 1.82 | 1.00 | 6.61 |
| 5730 | *PTGDS* | -2.41 | -1.89 | 1.00 | 6.61 |
| 9232 | *PTTG1* | 1.93 | 2.00 | 1.00 | 6.61 |
| 54517 | *PUS7* | 1.61 | 1.71 | 1.00 | 6.61 |
| 51109 | *RDH11* | -1.38 | -1.54 | 1.00 | 6.61 |
| 114822 | *RHPN1* | 2.04 | 1.65 | 1.00 | 6.61 |
| 6122 | *RPL3* | -1.68 | -1.56 | 1.00 | 6.61 |
| 29901 | *SAC3D1* | 1.76 | 1.91 | 1.00 | 6.61 |
| 23513 | *SCRIB* | 1.58 | 1.73 | 1.00 | 6.61 |
| 6510 | *SLC1A5* | -1.42 | -1.80 | 1.00 | 6.61 |
| 65010 | *SLC26A6* | 1.48 | 1.59 | 1.00 | 6.61 |
| 6591 | *SNAI2* | -1.99 | -1.89 | 1.00 | 6.61 |
| 6621 | *SNAPC4* | 1.69 | 1.97 | 1.00 | 6.61 |
| 8835 | *SOCS2* | -1.31 | -1.59 | 1.00 | 6.61 |
| 6659 | *SOX4* | 1.44 | 1.58 | 1.00 | 6.61 |
| 8677 | *STX10* | 1.66 | 1.53 | 1.00 | 6.61 |
| 23673 | *STX12* | -1.85 | -1.80 | 1.00 | 6.61 |
| 23336 | *SYNM* | -3.11 | -2.23 | 1.00 | 6.61 |
| 10460 | *TACC3* | 2.23 | 1.86 | 1.00 | 6.61 |
| 6876 | *TAGLN* | -2.52 | -1.99 | 1.00 | 6.61 |
| 84948 | *TIGD5* | 1.79 | 1.67 | 1.00 | 6.61 |
| 7083 | *TK1* | 1.77 | 1.62 | 1.00 | 6.61 |
| 25907 | *TMEM158* | -1.35 | -1.52 | 1.00 | 6.61 |
| 7169 | *TPM2* | -3.48 | -1.99 | 1.00 | 6.61 |
| 22974 | *TPX2* | 1.97 | 1.64 | 1.00 | 6.61 |
| 10024 | *TROAP* | 1.97 | 1.79 | 1.00 | 6.61 |
| 283989 | *TSEN54* | 1.48 | 1.62 | 1.00 | 6.61 |
| 7105 | *TSPAN6* | -1.41 | -1.76 | 1.00 | 6.61 |
| 7259 | *TSPYL1* | -1.79 | -1.66 | 1.00 | 6.61 |
| 29089 | *UBE2T* | 1.99 | 1.97 | 1.00 | 6.61 |
| 7349 | *UCN* | 1.80 | 2.04 | 1.00 | 6.61 |
| 10785 | *WDR4* | 1.67 | 1.61 | 1.00 | 6.61 |
| 197335 | *WDR90* | 1.13 | 1.54 | 1.00 | 6.61 |
| 23214 | *XPO6* | 1.75 | 1.62 | 1.00 | 6.61 |
| 79844 | *ZDHHC11* | 1.47 | 1.67 | 1.00 | 6.61 |
| 55205 | *ZNF532* | -1.38 | -1.64 | 1.00 | 6.61 |

Log_2_fold change>1.5 or <-1.5 and adjusted *P* value<0.001 were used to identify significantly differentially expressed genes between tumors and normal or benign tissues.

**Supplementary Table S4**. Prostate cancer cell line mappings among CCLE, LINCS, ChEMBL, and CTRP.

| CCLE | LINCS | ChEMBL | CTRP |
| --- | --- | --- | --- |
| 22RV1 | 22RV1 | 22RV1 | 22RV1 |
| - | BPH-1 | BPH-1 | - |
| DU145 | DU145 | DU145 | DU145 |
| - | - | DuPro | - |
| - | LNCaP | LNCaP | - |
| LNCaP clone FGC | - | LNCaP clone FGC | LNCaP clone FGC |
| - | - | LAPC4 | - |
| MDA PCa 2b | - | - | MDA PCa 2b |
| NCI-H660 | - | - | NCI-H660 |
| PC3 | PC3 | PC3 | PC3 |
| - | PC3.101 | - | - |
| - | PC3.311 | - | - |
| - | - | PrEC | - |
| PRECLH | - | - | PRECLH |
| VCAP | VCAP | - | VCAP |

CCLE, Cancer Cell Line Encyclopedia; LINCS, The Library of Integrated Network-Based Cellular Signatures; ChEMBL, a chemical database of bioactive molecules maintained by the European Bioinformatics Institute of the European Molecular Biology Laboratory; CTRP, The Cancer Therapeutics Response Portal

**Supplementary Table S5**. Performance of different methods used to compute RGES and sRGES.

| cell | Method | correlation coefficient |
| --- | --- | --- |
| Single cell lines* | RGES | 0.192 |
|  | Spearman | 0.153 |
|  | Pearson | 0.212 |
|  | Cosine | 0.223 |
| All cell lines^†^ | Median of RGES | 0.193 |
|  | Median of Pearson coefficient | 0.219 |
|  | Median of Spearman coefficient | 0.191 |
|  | Mean of RGES | 0.189 |
|  | Mean of Pearson coefficient | 0.218 |
|  | Mean of Spearman coefficient | 0.156 |
|  | Minimum of RGES | 0.279 |
|  | Minimum of Pearson coefficient | 0.158 |
|  | Minimum of Spearman coefficient | 0.220 |
|  | summarized RGES | 0.213 |
|  | summarized RGES using cell lines from the same lineage | 0.143 |
|  | summarized RGES using cell lines from different lineages | 0.188 |
|  | summarized RGES without weighting cell lines | 0.194 |
|  | summarized RGES using cell lines from the same lineage and without weighting cell lines | 0.199 |
|  | summarized RGES using cell lines from different lineages and without weighting cell lines | 0.165 |

*Correlation between reversal potency with drug efficacy in single cell lines. Reversal potency was measured using RGES, Spearman Correlation, Pearson Correlation and Cosine similarity. ^†^methods used to summarize the scores of reversal potency computed from metrics. Only expression of landmark genes was used.

**Supplementary Table S6**. Predicted drugs against castration resistant prostate cancer.

| Drug | Median IC_50_^*^ | Activity | Drug | Median IC_50_^*^ | Activity |
| --- | --- | --- | --- | --- | --- |
| amsacrine | 1.8000 | active | afatinib | 10.2044 | inactive |
| belinostat | 0.4500 | active | betulinin-acid | 37.0000 | inactive |
| bisindolylmaleimide-ix | 1.5849 | active | bicalutamide | 10.1395 | inactive |
| bortezomib | 0.0077 | active | bosutinib | 52.9261 | inactive |
| camptothecin | 0.3450 | active | caffeic-acid | 100.0000 | inactive |
| celastrol | 2.5000 | active | celecoxib | 48.0000 | inactive |
| colchicine | 0.0160 | active | chlorambucil | 127.8000 | inactive |
| dasatinib | 0.0623 | active | chloroquine | 32.8700 | inactive |
| digitoxin | 0.4400 | active | ciglitazone | 69.0000 | inactive |
| docetaxel | 0.0071 | active | doxazosin | 37.5550 | inactive |
| doxorubicin | 1.0650 | active | embelin | 11.7824 | inactive |
| elesclomol | 0.0200 | active | enzalutamide | 21.8700 | inactive |
| emetine | 0.0330 | active | erlotinib | 18.8750 | inactive |
| entinostat | 1.5000 | active | flufenamic-acid | 50.0000 | inactive |
| erbstatin-analog | 2.1628 | active | fluorouracil | 11.9500 | inactive |
| etoposide | 2.6000 | active | flutamide | 46.7000 | inactive |
| fenretinide | 5.5500 | active | gefitinib | 11.2500 | inactive |
| finasteride | 3.9000 | active | genistein | 47.2900 | inactive |
| floxuridine | 0.0692 | active | imatinib | 24.5594 | inactive |
| geldanamycin | 0.4300 | active | lenalidomide | 336.6622 | inactive |
| gemcitabine | 0.0356 | active | linsitinib | 38.8329 | inactive |
| irinotecan | 0.2000 | active | lorglumide | 100.0000 | inactive |
| lapatinib | 3.6800 | active | motesanib | 13.5068 | inactive |
| lestaurtinib | 1.5550 | active | navitoclax | 84.0430 | inactive |
| methotrexate | 0.0594 | active | nilotinib | 67.9994 | inactive |
| midostaurin | 9.7238 | active | nutlin-3 | 20.0400 | inactive |
| mitomycin-c | 0.3100 | active | olaparib | 152.0700 | inactive |
| mitoxantrone | 0.0071 | active | olomoucine | 81.0000 | inactive |
| mocetinostat | 1.3650 | active | pazopanib | 212.7033 | inactive |
| narciclasine | 0.0300 | active | piperine | 50.0000 | inactive |
| nocodazole | 2.1000 | active | prazosin | 67.4600 | inactive |
| obatoclax | 0.5154 | active | rofecoxib | 125.0000 | inactive |
| ouabain | 0.0430 | active | rosiglitazone | 18.1500 | inactive |
| paclitaxel | 0.0212 | active | rottlerin | 20.0000 | inactive |
| palbociclib | 5.4813 | active | rucaparib | 14.5342 | inactive |
| panobinostat | 0.0240 | active | selumetinib | 63.8670 | inactive |
| podophyllotoxin | 0.0340 | active | semaxanib | 50.0000 | inactive |
| saracatinib | 0.6200 | active | sorafenib | 15.0050 | inactive |
| sirolimus | 0.0100 | active | sunitinib | 16.3000 | inactive |
| tanespimycin | 0.2820 | active | tamoxifen | 10.0000 | active |
| temsirolimus | 0.0324 | active | tiratricol | 34.8000 | inactive |
| tipifarnib | 5.9375 | active | tranylcypromine | 100.0000 | inactive |
| topotecan | 0.1850 | active | tretinoin | 30.2773 | inactive |
| tozasertib | 3.1050 | active | troglitazone | 26.0000 | inactive |
| trichostatin-a | 1.8500 | active | umbelliferone | 111.6751 | inactive |
| triptolide | 0.0220 | active | ursolic-acid | 21.8957 | inactive |
| vinblastine | 0.0055 | active | veliparib | 127.4018 | inactive |
| vindesine | 0.0095 | active | xanthohumol | 10.6700 | inactive |
| vinorelbine | 0.0068 | active | - |  |  |
| vorinostat | 2.5350 | active | - |  |  |

^*^IC_50_, The half maximal inhibitory concentration

**Supplementary Table S7**. The top‐ranked drug combinations by a Target Inhibition Interaction using Maximization and Minimization Averaging algorithm

| Drug pairs | Sensitivity | Syn.add | Syn.multi | Syn.high | Targets |
| --- | --- | --- | --- | --- | --- |
| Lenalidomide + pazopanib | 0.880 | 0.721 | 0.874 | 0.777 | *APOE, ZFP36, ATMIN* |
| Olaparib + lenalidomide | 0.876 | 0.726 | 0.871 | 0.773 | *E2F2, APOE, ZFP36* |
| Nocodazole + lenalidomide | 0.871 | 0.682 | 0.862 | 0.768 | *MYL9, ZFP36, APOE* |
| Tipifarnib + lenalidomide | 0.871 | 0.682 | 0.862 | 0.768 | *MYL9, ZFP36, APOE* |
| Imatinib + lenalidomide | 0.871 | 0.682 | 0.862 | 0.768 | *MYL9, ZFP36, APOE* |
| Piperine + lenalidomide | 0.616 | 0.475 | 0.612 | 0.514 | *CENPE, ATMIN, APOE, ZFP36* |
| Floxuridine + lenalidomide | 0.613 | 0.478 | 0.610 | 0.511 | *CENPE, E2F2, ZFP36, APOE* |
| Umbelliferone + lenalidomide | 0.613 | 0.478 | 0.610 | 0.511 | *CENPE, E2F2, ZFP36, APOE* |
| Enzalutamide + lenalidomide | 0.613 | 0.478 | 0.610 | 0.511 | *CENPE, E2F2, ZFP36, APOE* |
| erbstatin-analog + sirolimus | 0.612 | 0.467 | 0.607 | 0.510 | *APOE, ATMIN, ZFP36* |
| erbstatin-analog + midostaurin | 0.612 | 0.467 | 0.607 | 0.510 | *APOE, ATMIN, ZFP36* |
| erbstatin-analog + lenalidomide | 0.612 | 0.467 | 0.607 | 0.510 | *ATMIN, APOE, ZFP36* |
| Sirolimus + caffeic-acid | 0.612 | 0.467 | 0.607 | 0.510 | *ATMIN, ZFP36, APOE* |
| Sirolimus + emetine | 0.612 | 0.467 | 0.607 | 0.510 | *ATMIN, ZFP36, APOE* |
| Sirolimus + narciclasine | 0.612 | 0.467 | 0.607 | 0.510 | *ATMIN, ZFP36, APOE* |
| Sirolimus + lenalidomide | 0.612 | 0.467 | 0.607 | 0.510 | *ATMIN, ZFP36, APOE* |
| Midostaurin + caffeic-acid | 0.612 | 0.467 | 0.607 | 0.510 | *ATMIN, ZFP36, APOE* |
| Midostaurin + emetine | 0.612 | 0.467 | 0.607 | 0.510 | *ATMIN, ZFP36, APOE* |
| Midostaurin + narciclasine | 0.612 | 0.467 | 0.607 | 0.510 | *ATMIN, ZFP36, APOE* |
| Midostaurin + lenalidomide | 0.612 | 0.467 | 0.607 | 0.510 | *ATMIN, ZFP36, APOE* |
| caffeic-acid + lenalidomide | 0.612 | 0.467 | 0.607 | 0.510 | *ATMIN, APOE, ZFP36* |
| Emetine + lenalidomide | 0.612 | 0.467 | 0.607 | 0.510 | *ATMIN, APOE, ZFP36* |
| Narciclasine + lenalidomide | 0.612 | 0.467 | 0.607 | 0.510 | *ATMIN, APOE, ZFP36* |
| Linsitinib + lenalidomide | 0.609 | 0.470 | 0.605 | 0.507 | *E2F2, ATMIN, APOE, ZFP36* |
| Rofecoxib + lenalidomide | 0.604 | 0.444 | 0.598 | 0.501 | *E2F2, MYL9, APOE, ZFP36* |
| Navitoclax + lenalidomide | 0.604 | 0.444 | 0.598 | 0.501 | *E2F2, MYL9, APOE, ZFP36* |
| Geldanamycin + lenalidomide | 0.600 | 0.416 | 0.592 | 0.498 | *MYL9, ZFP36, APOE* |
| Nocodazole + tipifarnib | 0.600 | 0.416 | 0.592 | 0.498 | *MYL9, APOE, ZFP36* |
| Nocodazole + imatinib | 0.600 | 0.416 | 0.592 | 0.498 | *MYL9, APOE, ZFP36* |
| nutlin-3 + lenalidomide | 0.600 | 0.416 | 0.592 | 0.498 | *MYL9, APOE, ZFP36* |
| Tipifarnib + imatinib | 0.600 | 0.416 | 0.592 | 0.498 | *MYL9, APOE, ZFP36* |
| Olaparib + pazopanib | 0.523 | 0.401 | 0.519 | 0.433 | *E2F2, APOE, ZFP36, ATMIN* |
| Nocodazole + pazopanib | 0.522 | 0.367 | 0.516 | 0.423 | *APOE, MYL9, ZFP36, ATMIN* |
| Tipifarnib + pazopanib | 0.522 | 0.367 | 0.516 | 0.423 | *APOE, MYL9, ZFP36, ATMIN* |
| Imatinib + pazopanib | 0.522 | 0.367 | 0.516 | 0.423 | *APOE, MYL9, ZFP36, ATMIN* |
| Olaparib + nocodazole | 0.520 | 0.373 | 0.515 | 0.422 | *E2F2, APOE, ZFP36, MYL9* |
| Olaparib + tipifarnib | 0.520 | 0.373 | 0.515 | 0.422 | *E2F2, APOE, ZFP36, MYL9* |
| Olaparib + imatinib | 0.520 | 0.373 | 0.515 | 0.422 | *E2F2, APOE, ZFP36, MYL9* |
| erbstatin-analog + pazopanib | 0.491 | 0.354 | 0.487 | 0.391 | *ATMIN, APOE, ZFP36* |
| caffeic-acid + pazopanib | 0.491 | 0.354 | 0.487 | 0.391 | *ATMIN, APOE, ZFP36* |
| Emetine + pazopanib | 0.491 | 0.354 | 0.487 | 0.391 | *ATMIN, APOE, ZFP36* |
| Narciclasine + pazopanib | 0.491 | 0.354 | 0.487 | 0.391 | *ATMIN, APOE, ZFP36* |
| Methotrexate + lenalidomide | 0.479 | 0.348 | 0.476 | 0.378 | *CENPE, E2F2, ATMIN, APOE, ZFP36* |
| Tozasertib + lenalidomide | 0.479 | 0.348 | 0.476 | 0.378 | *CENPE, E2F2, ATMIN, APOE, ZFP36* |
| Fluorouracil + lenalidomide | 0.475 | 0.329 | 0.470 | 0.373 | *CENPE, E2F2, MYL9, ZFP36, APOE* |
| Lapatinib + lenalidomide | 0.475 | 0.329 | 0.470 | 0.373 | *CENPE, E2F2, MYL9, ZFP36, APOE* |
| Lenalidomide + erlotinib | 0.475 | 0.329 | 0.470 | 0.373 | *APOE, ZFP36, CENPE, E2F2, MYL9* |
| Lenalidomide + bosutinib | 0.472 | 0.323 | 0.467 | 0.370 | *APOE, ZFP36, E2F2, ATMIN, MYL9* |
| Sirolimus + pazopanib | 0.465 | 0.325 | 0.461 | 0.362 | *ATMIN, ZFP36, APOE, ATMIN* |
| midostaurin + pazopanib | 0.465 | 0.325 | 0.461 | 0.362 | *ATMIN, ZFP36, APOE, ATMIN* |

Scores were calculated by a TIMMA-R package; Syn.add, Synergic addictive score; Syn.multi, Synergic multiplicative score; Syn.high, Synergic highest agent
